# Factors Associated with Negative Direct Sputum Examination in Asian and African HIV-Infected Patients with Tuberculosis (ANRS 1260)

**DOI:** 10.1371/journal.pone.0021212

**Published:** 2011-06-23

**Authors:** Loïc Chartier, Chanthy Leng, Jean-Marie Sire, Odile Le Minor, Manil Saman, Raymond Bercion, Lila Rahalison, Arnaud Fontanet, Yves Germany, Pierre L'Her, Charles Mayaud, Muriel Vray

**Affiliations:** 1 Institut Pasteur, Paris, France; 2 Institut Pasteur, Phnom Penh, Cambodge; 3 Institut Pasteur, Dakar, Sénégal; 4 Hôpital Principal, Dakar, Sénégal; 5 Institut Pasteur, Tananarive, Madagascar; 6 Conservatoire National des Arts et Métiers, Paris, France; 7 International Network of Institut Pasteur, Paris, France; 8 Hôpital militaire, Percy, France; 9 Hôpital Tenon, Paris, France; San Francisco General Hospital, University of California San Francisco, United States of America

## Abstract

**Objective:**

To identify factors associated with negative direct sputum examination among African and Cambodian patients co-infected by *Mycobacterium tuberculosis* and HIV.

**Design:**

Prospective multicenter study (ANRS1260) conducted in Cambodia, Senegal and Central African Republic.

**Methods:**

Univariate and multivariate analyses (logistic regression) were used to identify clinical and radiological features associated with negative direct sputum examination in HIV-infected patients with positive *M. tuberculosis* culture on Lowenstein-Jensen medium.

**Results:**

Between September 2002 and December 2005, 175 co-infected patients were hospitalized with at least one respiratory symptom and pulmonary radiographic anomaly. Acid-fast bacillus (AFB) examination was positive in sputum samples from 110 subjects (63%) and negative in 65 patients (37%). Most patients were at an advanced stage of HIV disease (92% at stage III or IV of the WHO classification) with a median CD4 cell count of 36/mm^3^. In this context, we found that sputum AFB negativity was more frequent in co-infected subjects with associated respiratory tract infections (OR = 2.8 [95%CI:1.1–7.0]), dyspnea (OR = 2.5 [95%CI:1.1–5.6]), and localized interstitial opacities (OR = 3.1 [95%CI:1.3–7.6]), but was less frequent with CD4≤50/mm^3^ (OR = 0.4 [95%CI:0.2–0.90), adenopathies (OR = 0.4 [95%CI:0.2–0.93]) and cavitation (OR = 0.1 [95%CI:0.03–0.6]).

**Conclusions:**

One novel finding of this study is the association between concomitant respiratory tract infection and negative sputum AFB, particularly in Cambodia. This finding suggests that repeating AFB testing in AFB-negative patients should be conducted when broad spectrum antibiotic treatment does not lead to complete recovery from respiratory symptoms. In HIV-infected patients with a CD4 cell count below 50/mm3 without an identified cause of pneumonia, systematic AFB direct sputum examination is justified because of atypical clinical features (without cavitation) and high pulmonary mycobacterial burden.

## Introduction

*M. tuberculosis*/HIV co-infection is a major public health issue in developing countries. Although most co-infections occur in sub-Saharan Africa [1], [2], the number of co-infected patients has increased considerably in South-East Asia [1], [3]. In populations with a high prevalence of HIV infection, tuberculosis is a major cause of morbidity and mortality [1], particularly in Africa [4] and Asia [5]. Finally, tuberculosis, by facilitating HIV replication, accelerates progression to AIDS [6].

The immunosuppression induced by HIV infection increases the risk of developing tuberculosis [7]. In addition, the clinical expression of tuberculosis is modified in patients with severe immunodepression [1], [6]. Tuberculosis is generally easier to diagnose early in the course of HIV infection, owing to its classical expression (such as cavitation), and AFB examination is therefore routinely performed. In the later stages of HIV disease, clinical signs are more varied because of unusual clinical and radiological features. As a result, AFB examination is often delayed, and sputum smears may be negative particularly in patients without cavitation [1], [8]. In patients with severe immunosuppression, the mycobacterial burden may be very high and disseminated. For this reason, Mycobacterium tuberculosis can be found in sputum, blood and others organs (Brenda E. Jones et al [9]).

The reference diagnostic method for pulmonary tuberculosis is culture of *Mycobacterium tuberculosis* (MT) on specific Lowenstein-Jensen medium. However, culture is rarely available in developing countries and takes between 6 and 8 weeks, limiting its use to a second-line diagnostic examination [10]. Direct examination of sputum for the presence of AFB is currently the most useful diagnostic tool, since it is simple, rapid, cheap and highly specific [11], [12]. Unfortunately, its sensitivity is highly variable, especially in patients with AIDS [12].

AFB-negative tuberculosis is less of a public health problem [10] because it is less contagious, even though this form of tuberculosis is still responsible for 15 to 20% of cases of human-human transmission [13]. In individual patients coinfected by HIV, AFB-negative tuberculosis is associated with a high mortality rate, because of delayed access to antituberculous treatment and the high degree of underlying immunodepression [14]. Thus, AFB-negative patients pose a therapeutic dilemma: deciding to treat on clinical grounds alone may mask the real cause of clinical manifestations, carries a risk of unnecessary toxicity and can interfere with antiretroviral therapy [2], [15], yet failure to treat carries a risk of death from tuberculosis and of persistent infectivity [13].

Several studies have identified factors predictive of tuberculosis in AFB-negative HIV-infected patients [16], [17], [18], [19]. However, few have focused on clinical and radiological characteristics associated with AFB negativity in coinfected patients [20], [21], and few have included in-depth search for etiological agents of respiratory symptoms by means of bronchio-alveolar lavage (BAL). Furthermore, the epidemiological context differs from country to country, and predictive factors can also vary.

The aim of this work was to identify factors associated with negative AFB direct sputum examination among African and Cambodian patients coinfected by *M. tuberculosis* and HIV.

## Methods

### Study population

Data were collected as part of a multicenter study (ANRS1260) conducted in Africa (Senegal and Central African Republic (CAR) and Southeast Asia (Cambodia and Vietnam). This prospective study was principally designed to document the clinical and radiological manifestations and etiologies of pulmonary disease in HIV-infected patients with AFB-negative on direct sputum examination [22]. The data collection and laboratory methods are described in detail in the princeps article [22]. This study was approved by the Senegal Health Research National Council, the National Ethics Committee for Health Research in Cambodia, and Scientific Committee responsible for validation protocols and study results in Central African Republic.

Between September 2002 and December 2005, patients were enrolled in this study, with their written consent, if they were HIV-infected, at least 18 years old, and had at least one respiratory symptom (cough with or without expectoration, chest pain, haemoptysis and/or dyspnea) and at least one radiological pulmonary abnormality (diffuse or localized opacities, cavitation, and/or mediastinal adenopathies).

In Senegal and Cambodia, all HIV-infected patients entered an antiretroviral program. In CAR, however, antiretroviral treatment was not available.

This analysis focused on subjects recruited in Cambodia, Senegal and Central African Republic, where patient management and data collection were the same for patients with positive and negative direct sputum examination [22].

According to the protocol [22], three sputum samples for AFB detection were collected before further invasive investigations, unless invasive methods were immediately necessary because of severe lung disease. As a result, a single sputum sample was collected from most Cambodian patients, whose initial lung disease tended to be more severe, while two or three samples were collected from African patients. Cambodian patients underwent earlier BAL than African patients for the following reasons: severity of hypoxemia, high frequency of diffuse opacities, failure of antibiotic treatment and high frequency of *pneumocystis jiroveci.*

### Data collection

The following data were analyzed: i) general characteristics (age, sex, country) and clinical features (body mass index (BMI) (kg/m^2^), headache, diarrhea, dyspnea, hemoptysis, cough duration, stage of HIV infection (WHO classification), history of tuberculosis and prophylaxis with trimethoprim-sulfamethoxazole (TMP-SMX) >1 month), ii) biological data (CD4 lymphocyte count, AFB detection in sputum by direct examination and culture, results of BAL microbiological examination), anemia (hemoglobin <13 g/dl in men and <12 g/dl in women) and prior knowledge of HIV serostatus. The chest radiographs were examined by local physicians and by experts, as described in the princeps article [20]. Localized opacities were recorded as interstitial or alveolar. Blinded to the microbiological and clinical findings, two French experts in pulmonary medicine read the radiographs. In case of disagreement, the local physicians and the experts reached a consensus.

### Definition of definite pulmonary tuberculosis

Culture on Lowenstein-Jensen medium is the reference method. Thus, patients in whom *M. tuberculosis* was isolated by culture in sputum, bronchial fibroaspiration products or BAL fluid were considered to have definite pulmonary tuberculosis.

Tuberculosis was ruled out only when none of respiratory samples were culture positive.

This analysis focuses solely on patients with definite (culture-positive) tuberculosis. Among these patients, all those with at least one positive sputum sample on direct examination were considered AFB-positive and all subjects in whom all sputum samples (one to three) were negative by direct examination were considered AFB-negative.

### Definition of definite bacterial, parasitic, or yeast lung co-infection

- *Pneumocystis jiroveci* pneumonia (PCP): the presence of the pathogen in induced sputum or BAL fluid.
- Fungal pneumonia: positive direct examination and/or culture of fiberoptic specimen and a compatible clinical outcome.
- Bacterial pneumonia: positive blood culture or positive culture of pyogenic bacteria from pleural effusion or positive quantitative culture of a fiberoptic specimen with validated cutoffs [≥10^5^CFU/ml (bronchial aspirate) or ≥10^4^CFU/ml (BAL)] and a compatible clinical outcome.
- Pneumonia due to atypical mycobacteria: positive culture of fiberoptic specimen and no other pathogens isolated, and a compatible clinical outcome.
- Pulmonary strongyloidosis: *Strongyloides stercoralis* larvae in fiberoptic specimen and no other pathogens, and a compatible clinical outcome

Research on associated respiratory tract infections was conducted in all patients, even if investigative methods differed.

### Definition of probable bacterial lung co-infection

Positive quantitative culture of pyogenic bacteria from sputum, with validated cutoff (≥10^7^ CFU/ml) and a compatible clinical outcome; or positive quantitative culture of pyogenic bacteria from fibroaspirate or BAL specimen, without validated cutoffs but with a compatible clinical outcome.

### Statistical methods

The patients' characteristics were analyzed as medians and interquartile range (IQR) for continuous variables and percentages for discrete variables. Univariate analysis was based on the Chi2 test or Fisher's exact test for discrete variables. Continuous variables were compared by analysis of variance or Student's *t* test if the distribution was normal, and otherwise with the Kruskal-Wallis or Mann and Whitney test. For multivariate analysis, quantitative variables were categorized around the median or the clinical threshold (SaO2, CD4 cell count, and cough duration).

All baseline variables associated with AFB-negative tuberculosis in univariate analysis (p <0.25) were included in a backward stepwise logistic regression model. Interactions between factors associated with AFB negativity and the participating region (Cambodia and the two African countries) were tested. In order to account for the specificities of each region, the factor “region” was retained in the final model, even though it was not significant. The likelihood ratio method was used for significance testing. A p value of <0.05 was considered to denote statistical significance. Multiple imputations for the CD4 cell count missing values were performed by using variables from logistic regression model (country, radiological characteristics (mediastinal adenopathies, cavitation and localized interstitial) and clinical characteristics (dyspnea and more than one pathogen)).

Data were analyzed with STATA software version 11.0 (Stata Corporation, College Station, Texas).

## Results

### Description of the population

**Figure 1:**
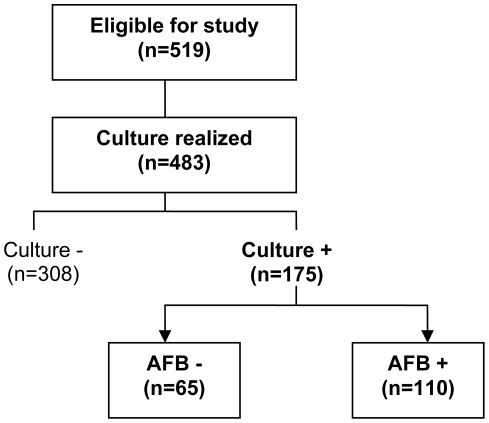
Distribution of AFB-positive and AFB-negative patients with definite tuberculosis in Cambodia and Africa.

Between September 2002 and December 2005, 175 HIV-infected patients with definite tuberculosis were included in this analysis (Fig. 1); 110 patients (63%) were AFB+ and 65 (37%) were AFB-. Owing to the severity of the pulmonary disease, AFB was performed in only one sputum sample in 93% of patients in Cambodia, compared to 12% and 3% of patients in Senegal and CAR.

Most cases of AFB-positive tuberculosis (89%) were diagnosed on the first sputum sample, including in Africa (79%), where three samples per patient were routinely examined.

Fiberoptic bronchoscopy with BAL was performed in 76 patients. Few cases of AFB-positive (n = 26, 24%) tuberculosis underwent bronchial fibroaspiration or BAL compared to AFB-negative patients (n = 50, 77%).

The main characteristics of the study population are summarized in table 1. Most patients were young adults (median age 34 years), with anemia (98%, median hemoglobin level 7.6 g/dl (6.2–9.4)), advanced stage of HIV disease (92% were at stage III (64%) or IV (28%) of the WHO classification), and a very low median BMI (16.9 kg/m^2^ (14.9–18.8)). The median CD4 cell count was 36/mm3, and 58% of the population had counts below 50 CD4/mm3, reflecting severe immunosuppression. Less than half the patients (43%) were aware of their HIV serostatus before enrollment. Of these, 14% had been on antiretroviral therapy for more than 15 days and 26% had been taking TMP-SMZ prophylaxis for more than a month. Few had a history of tuberculosis (14%). Most patients (85%) received empirical treatment.

**Table 1:** Baseline characteristics of the study population.

Parameter, n (%)	Cambodia	Senegal	Central African Republic	All	p value*
	N = 84	N = 25	N = 66	N = 175	
Number of subjects (% males)	57 (68)	12 (48)	22 (33)	91 (52)	<0.001
Age (years)**	34 (29–42)	36 (33–41)	32 (27–39)	34 (28–41)	0.084
Number of AFB-negative subjects	27 (32)	9 (36)	29 (44)	65 (37)	0.33
AFB+ on the					0.002
1st sputum sample	56 (98)	11 (69)	31 (84)	98 (89)	
2^nd^ or 3^rd^ sputum sample	1 (2)	5 (31)	6 (16)	12 (11)	
Antibiotics before admission, Yes	35 (78)	6 (27)	22 (33)	63 (47)	<0.001
Body Mass Index (BMI)** (kg/m^2^)	17.0 (14.8–18.8)	16.6 (14.2–18.7)	16.7 (15.4–18.4)	16.9 (14.9–18.8)	0.75
AIDS classification (WHO)					<0.001
Stage II	1 (1)	6 (27)	6 (10)	13 (8)	
Stage III	47 (56)	16 (73)	44 (73)	107 (64)	
Stage IV	36 (43)	0 (0)	10 (17)	46 (28)	
CD4 cell count (/mm^3^)**	18 (6–43)	36 (16–66)	177 (56–349)	36 (12–145)	<0.001
Hemoglobin (g/dl) **	8.1 (6.5–10.1)	7 (5.6–7.7)	7.5 (6–9.4)	7.6 (6.2–9.4)	0.044
Anemia, Yes	78 (98)	24 (96)	58 (98)	160 (98)	0.65
Resting oxygen saturation (SaO^2^) **	92 (90–96)	98 (97–99)	96 (92–98)	94 (90–97)	<0.001
Known HIV serostatus on admission, Yes	54 (64)	14 (56)	8 (12)	76 (43)	<0.001
- ARV for >15 days	8/54 (15)	2/14 (14)	1/8 (13)	11/76 (14)	0.99
- TMP-SMZ prophylaxis for >30 days	15/54 (28)	1/14 (7)	4/8 (50)	20/76 (26)	0.065
History of tuberculosis, Yes	13 (16)	2 (8)	9 (14)	24 (14)	0.62
More than one pathogen, Yes	14 (17)	4 (16)	9 (14)	27 (15)	0.88

*Global comparison among the three countries.

**Median (Q1, Q3).

In 15% of cases, one or more other pathogens were found in a valid sample (78% from fibroaspirate or BAL specimen and 22% from sputum), in addition to *M. tuberculosis*. In the 14 Cambodian patients concerned, the pathogens were *Pneumocystis jiroveci* (n = 6), *Staphylococcus aureus* (n = 2), *Pseudomonas aeruginosa* (n = 2), *Streptococcus pneumoniae* (n = 1), *Escherichia coli* (n = 1), *Strongyloides stercoralis* (n = 2) and/or *Cryptococcus neoformans* (n = 2). In the 13 African patients concerned, the pathogens were *Staphylococcus aureus* (n = 5), *Pseudomonas aeruginosa* (n = 3), *Streptococcus pneumoniae* (n = 3), *Klebsiella pneumoniae* (n = 2), *Cryptococcus neoformans* (n = 1) and/or *Mycobacterium intracellulare* (n = 1).

Cambodian patients differed significantly from African patients, with more frequent antibiotic therapy before hospital admission (78% in Cambodia, 27% and 33% in Senegal and CAR), more frequent WHO stage IV HIV infection (43% in Cambodia, 17% in CAR and 0% in Senegal), a higher median hemoglobin level (8.1 (6.5–10.1) g/dl in Cambodia, 7.0 g/dl (5.6–7.7) in Senegal), and lower oxygen saturation (92% (90–96) in Cambodia versus 96% (92–98) in CAR and 98% (97–99) in Senegal).

The median CD4 cell count was higher in CAR than in the other two countries (177/mm^3^ (56–349) in CAR, 18/mm^3^ (6–43) in Cambodia and 36/mm^3^ (16–66) in Senegal). Fewer patients in CAR were aware of their HIV serostatus (12%, compared to 56% and 64% in Senegal and Cambodia).

### Univariate analysis

Table 2 shows the results of univariate analysis comparing general, clinical, biological and radiological characteristics between AFB+ and AFB- patients.

**Table 2:** Factors associated with sputum smear negativity by univariate and multivariate analysis.

Characteristics	AFB+	AFB−	Univariate analysis		Multivariate analysis	
	(n = 110)	(n = 65)	OR* (CI_95%_)	P	OR* (CI_95%_)	P
	N (%)	N (%)		(p≤0.25)		(p≤0.05)
**General characteristics**						
Region						
Africa (Senegal, CAR)	53 (48)	38 (58)	1		1	
Asia (Cambodia)	57 (52)	27 (42)	0.7 (0.4–1.2)	0.21	0.6 (0.3–1.4)	0.24
**Clinical characteristics**						
Dyspnea, Yes	63 (57)	43 (66)	1.5 (0.8–2.8)	0.25	2.5 (1.1–5.6)	0.031
Cough ≥21 days	62 (57)	28 (46)	0.6 (0.3–1.2)	0.15		
More than one pathogen	12 (11)	15 (23)	2.5 (1.1–5.6)	0.035	2.8 (1.1–7.0)	0.033
**Biological characteristics**						
CD4 (/mm^3^)						
>50	33 (30)	32 (49)	1		1	
≤50	65 (59)	25 (38)	0.4 (0.2–0.8)	0.007	0.4 (0.2–0.90)	0.028
Missing data	12 (11)	8 (12)	0.7 (0.3–1.9)	0.47		
**Radiological characteristics**						
Localized interstitial, Yes	12 (11)	18 (28)	3.1 (1.4–7)	0.006	3.1 (1.3–7.6)	0.014
Mediastinal adenopathies, Yes	61 (55)	26 (40)	0.5 (0.3–1)	0.049	0.4 (0.2–0.93)	0.031
Cavitation, Yes	18 (16)	2 (3)	0.2 (0.04–0.7)	0.017	0.1 (0.03–0.6)	0.012
Retraction, Yes	11 (10)	0 (0)	0.1 (0 –0.6)	0.007		

*OR: Odds Ratio.

AFB negativity was more likely in patients with localized interstitial radiological anomalies (OR = 3.1 [95%CI:1.4–7]) and intercurrent bacterial or parasitic infections (OR = 2.5 [95%CI:1.1–5.6]). In contrast, CD4 cell counts ≤50/mm^3^ (OR = 0.4 [95%CI:0.2–0.8]), adenopathies (OR = 0.54 [95%CI:0.3–1]), cavitation (OR = 0.16 [95%CI:0.04–0.7]) and retraction (OR = 0.10 [95%CI:0–0.6]) were associated with a lower risk of AFB negativity.

### Multivariate analysis

Three factors were independently associated with AFB negativity, namely dyspnea (OR = 2.5 [95%CI:1.1–5.6]), localized interstitial radiological abnormalities (OR = 3.1 [95%CI:1.3–7.6]) and intercurrent bacterial or parasitic infections (OR = 2.8 [95%CI:1.1–7.0]). Three factors were independently associated with a lower risk of AFB negativity, namely adenopathies (OR = 0.4 [95%CI: 0.2–0.93]), cavitation (OR = 0.1 [95%CI: 0.03–0.6]) and CD4 fewer than 50 /mm^3^ (OR = 0.4 [95%CI:0.2–0.90]) (Table 2). The findings remain consistent with a multivariate model that does not account for multiple imputations.

Given the large differences between the Cambodian and African patients, the same analysis was repeated separately in the two subpopulations. Different risk factors were identified. In Cambodia, three factors were independently associated with a higher risk of AFB negativity, namely dyspnea (OR = 6.5 [95%CI:1.2–34.9]), localized interstitial radiological abnormalities (OR = 7.5 [95%CI:2.2–25.9]) and intercurrent bacterial, fungal or parasitic infections (OR = 3.8 [95%CI:0.95–15.4]), and none was independently associated with a lower risk of AFB negativity. In Africa, none was independently associated with a higher risk of AFB negativity but two factors were independently associated with a lower risk of AFB negativity, namely cavitation (OR = 0.08 [95%CI: 0.01–0.72]) and cough lasting ≥21 days (OR = 0.3 [95%CI:0.13–0.86]).

## Discussion

The patients analyzed here were all enrolled in the ANRS 1260 study. All had HIV infection and definite pulmonary tuberculosis.

Our study compared clinical and radiological features in a significant number of HIV-coinfected AFB+ and AFB- patients with tuberculosis. Most patients were in an advanced stage of HIV disease (92% were at stage III or IV of the WHO classification) with a median CD4 cell count of 36/mm^3^, reflecting severe immunosuppression. In this context, we found that sputum AFB negativity was more frequent in co-infected subjects with dyspnea, localized interstitial opacities, an intercurrent bacterial, fungal or parasitic respiratory tract infection, with CD4 >50/mm^3^, no adenopathies and no cavitation. Analyses by region showed that associations were going in the same direction for all the factors statistically significant in the final model. However, due to limitation of power, only dyspnea, localized interstitial opacities, and intercurrent respiratory tract infection were statistically significant in Cambodia, whereas only absence of cavitation was statistically significant in Africa. Whether these latter findings reflect differences by region, or random fluctuations, cannot be tested formally due to the lack of power. These diverse results probably reflect the highly variable radiological and clinical expression, as well as the intrapulmonary mycobacterial burden, all of which depend on the degree of immunodepression. Indeed, severely immunodepressed patients often have unusual clinical and radiological features, such as more discreet cough, normal chest X ray, miliary opacities or diffuse infiltrate, no cavitation, frequent mediastinal adenopathies and pleuritis, a high bacillary burden, and frequent association with another respiratory tract infection [1], [8]. Our findings underscore that AFB-negative tuberculosis in HIV-infected patients can be associated with less-clearcut radiological and clinical signs, including: i) dyspnea suggestive of PCP, ii) the absence of mediastinal adenopathies (depriving the clinician of a major radiological sign suggestive of tuberculosis) and iii) symptoms or signs resulting of intercurrent respiratory tract infection (with the risk of failing to recognize associated tuberculosis in the absence of unexplained atypical clinical data or clinical progression despite adequate treatment).

The CD4 cell count plays an important role in the clinical manifestations of tuberculosis in HIV-infected patients. Some studies have reported that sputum smears are frequently negative for AFB in patients with advanced HIV disease [1], [8]. Conversely, a CD4 cell count below 50/mm^3^ was more common in our study in patients with AFB positivity than in patients with AFB negativity. This finding, which concurs with other studies and particularly with Brenda E. Jones et al.'s study [9], can be explained by the increased degree of intrapulmonary mycobacterial burden in parallel with the degree of immunosuppression. In our study, the vast majority of AFB-negative cavitation observed in patients with advanced immunosuppression results from necrotizing bacterial infections, not from tuberculosis.

One of the most interesting findings of the study is the negativity of AFB in the presence of bacterial, parasitic, or fungal lung co-infection. This finding remained statistically significant in multivariate analysis, suggesting that it is independent of potential confounding variables such as the level of immunosuppression. One may speculate that the presence of other infectious agents may inhibit the production of mycobacteria.

Another hypothesis could be that co-infected patients are less likely to produce sputum positive because they have more severe clinical symptoms, such as dyspnea or high fever.

Interestingly, this finding may have important implications for the management of HIV-infected patients who are AFB-negative but who nevertheless have symptoms suggestive of tuberculosis. Indeed, current WHO guidelines suggest that such patients be treated with two weeks of first-line antibiotics to rule out bacterial infection. In patients who have both tuberculosis and bacterial lung infections, this short antibiotic treatment would lead to immediate improvement, because of its overall efficacy against *Streptococcus pneumoniae* or *Hemophilus influenza,e* or its partial efficacy against *Staphylococcus species* or *Enterobacteriacae*. Consequently, in the absence of full and durable recovery following antibiotic treatment, it would be important to repeat AFB testing [23] or to use the MTB/RIF test (*Mycobacterium tuberculosis* (MTB) and resistance to rifampin (RIF)) [24]. Indeed, this test is easy to use, inexpensive and available in developing countries. It is possible that *M.tuberculosis* would be easier to detect once the bacterial co-infection has been treated, if indeed the latter was responsible for AFB-negativity. Another strategy would be to use empirical tuberculosis treatment in conjunction with antiviral therapy in a subset of patients with advanced immuno-deficiency who are living in very high TB burden communities [25].

Our study has several limitations. First, the number of coinfected patients with AFB- negativity was relatively small. Second, some clinical data, such as the interval between symptom onset and hospitalization, the presence of fever on admission [22] and the Karnofsky score were too unreliable for meaningful analysis. Third, not all of our patients had three sputum smear examinations. Finally, the lack of radiological controls at 21 days rules out any analysis of the part played by tuberculosis in the radiological anomalies observed in patients with intercurrent infections.

One novel finding of this study is the association between concomitant respiratory tract infection and negative sputum AFB, particularly in Cambodia. This finding suggests repeating AFB testing in AFB-negative patients, when broad spectrum antibiotics treatment is not followed by complete recovery of the respiratory symptoms. In HIV-infected patients with a CD4 cell count below 50/mm^3^ without an identified cause of pneumonia, systematic AFB direct sputum examination is justified because of atypical clinical features (without cavitation) and high pulmonary mycobacterial burden.
